# Pathophysiology and Clinical Implications of Cognitive Dysfunction in Fibromyalgia

**DOI:** 10.7759/cureus.19123

**Published:** 2021-10-29

**Authors:** Weaam Ibraheem, Simon Mckenzie, Victory Wilcox-Omubo, Mohamed Abdelaty, Sandra E Saji, Rosemary Siby, Wafaa Alalyani, Jihan A Mostafa

**Affiliations:** 1 Medical Research, California Institute of Behavioral Neurosciences & Psychology, Fairfield, USA; 2 Psychiatry, California Institute of Behavioral Neurosciences & Psychology, Fairfield, USA

**Keywords:** fibromyalgia, cognitive dysfunction, fibro-fog, pathophysiology, risk factors

## Abstract

Cognitive dysfunction is a complaint of many patients diagnosed with fibromyalgia. Although the main symptoms of the disease are fatigue, widespread musculoskeletal pain, poor sleep quality, and tenderness points, the cognitive symptoms can be more distressing than the pain itself, and negatively affect their lives; however, many healthcare professionals underestimate these cognitive complaints and it is still one of the least researched topics. Proper management of these symptoms at an early stage may have a great impact to improve the mental health, physical function, and overall health of these patients. Hence, this traditional review aimed to look at the previous body of literature in PubMed in the past five years to address the pathophysiology of the cognitive dysfunction in fibromyalgia patients, to find the risk factors of cognitive dysfunction in these patients, to discover the recent modalities for treatment, and to figure out the clinical implications and recent recommendations by researchers on screening, diagnosis, and management of fibromyalgia and its cognitive dysfunction symptoms.

This review has shown the various mechanisms of cognitive dysfunction. Some mechanisms are related to disease symptomologies, such as excessive pain perception, and others are related to hormonal and metabolite changes in the brain. Tobacco smoking and high body mass index showed an inverse impact on cognitive dysfunction and quality of life in fibromyalgia. Other risk factors and clinical implications were discussed in detail.

## Introduction and background

"What most people don’t understand is that pain itself can cause harmful side effects and can affect concentration and mental clarity just as profoundly as any drug." Dr. Scott Strassels [1].

This is what fibromyalgia patients suffer from with their pain and thinking every day, and many people do not realize that pain in fibromyalgia is not the only disturbing symptom, there is another aspect of the disease that is affecting their cognitive functions and can be severe enough to affect their lives so dramatically and need to be addressed and managed properly in a timely fashion.

Fibromyalgia is a disorder that commonly affects females than males, characterized by chronic generalized musculoskeletal pain and psychosomatic symptoms like fatigue, headache, sleep disturbance, anxiety, mood changes, and cognitive dysfunction. Fibro fog is a term used for cognitive dysfunction symptoms in fibromyalgia, and these symptoms include memory impairment, reduced mental clarity, and reduced attention and focus [2].

One meta-analysis study in 2018 has reviewed 37 case-control studies with 964 fibromyalgia patients compared to the age-matched control group (n = 1,025). Tyler et al. found that the self-reported cognitive symptoms are associated with reduced performance of cognitive functions tests compared to the control group, which indicates that cognitive dysfunction is also an objective tested symptom [3].

In another meta-analysis of 23 case-control studies with 2,096 participants and with an application of neurophysiological tests, Wu et al. found that fibromyalgia affects various domains of cognitive function, and the large effect sizes were found in learning, memory, attention, and psychomotor speed, and medium effect sizes were found in executive function and working memory. Also, it was shown that anxiety and depression scores are associated with the effect size of group differences in cognitive dysfunction and this may partially explain the heterogeneity across studies [4].

It has been proposed that fibromyalgia and its dyscognition are related to hippocampus dysfunction [5], which is a component of the limbic system that has a great role in memory and cognition, and it is also known to have a role in nociception (the detection of painful stimuli). This mechanism depends on the activation of N-methyl-D-aspartate (NMDA) subtype glutamate receptors, which can be largely activated by stress-related hormones. An increase of glucocorticoids after prolonged stress exposure and excessive NMDA activation by excitatory neurotransmission will end up in hippocampal dysfunction and atrophy [5].

There are other proposed pathophysiological mechanisms that have been studied recently. Some studies have focused on the relationship between stress maladaptation, level of cortisone, and developing cognitive dysfunction [6], and some research studies have studied how the brain is acting during the cognitive function tests in fibromyalgia-fibro fog patients.

Risk factors of cognitive dysfunction in fibromyalgia patients were also another target for researchers to discuss in the past five years like smoking [7], depression [8], as well as the other new modalities of fibro fog treatment.

This review is planned to address the pathophysiology of cognitive function decline in fibromyalgia patients, risk factors of developing cognitive dysfunction in fibromyalgia, the impact of external environment on cognitive function in fibromyalgia, new modalities of non-invasive brain stimulation in the management of fibromyalgia cognitive dysfunction, implications of fibro fog in clinical practice, testing, and treatment of fibromyalgia.

**Figure 1:**
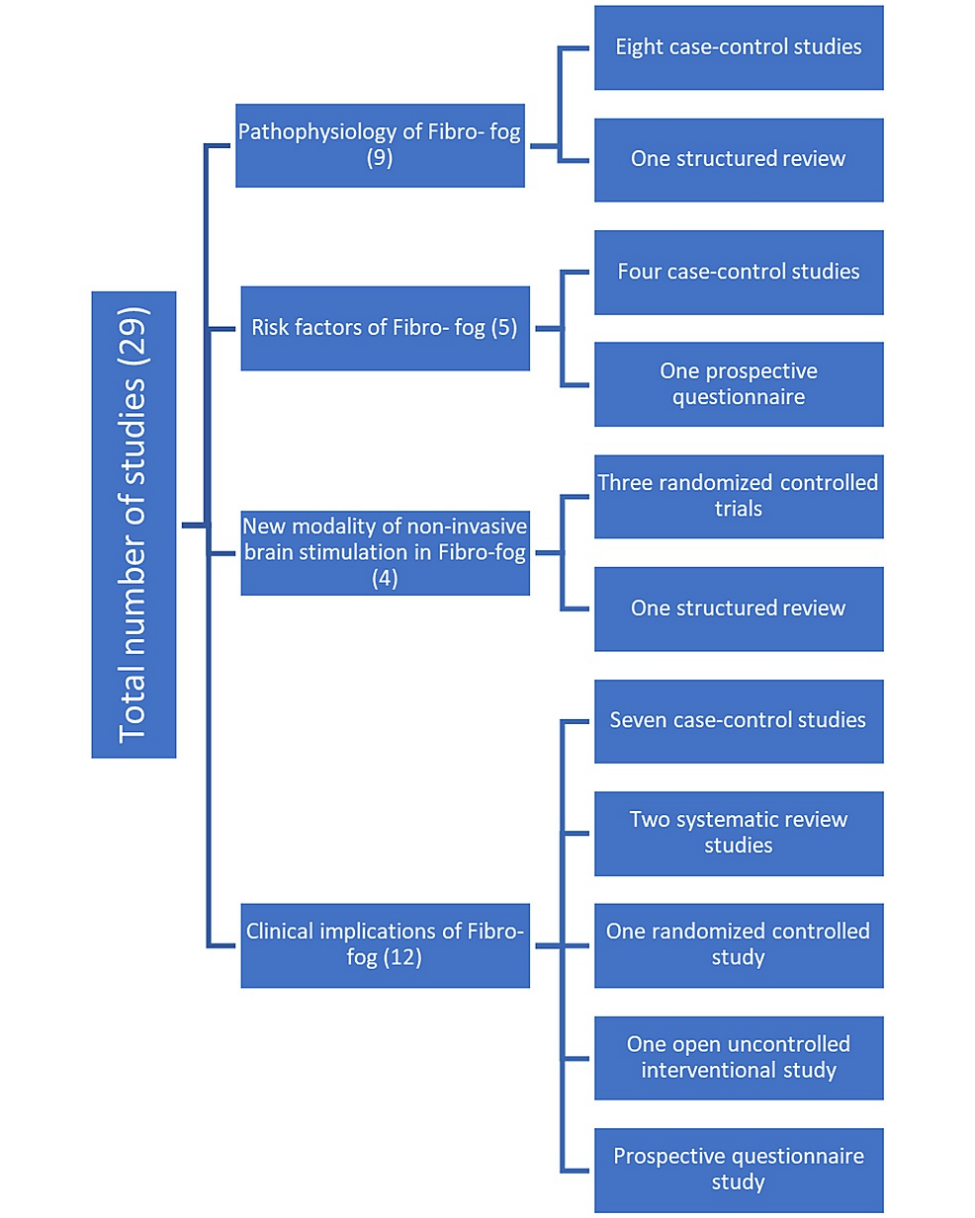
Total number of studies in the review.

The search was performed using the following regular keywords: "fibromyalgia," "chronic widespread pain," "fibro fog," "pathophysiology," "risk factors," "memory loss," "attention deficit," "cognitive disorders," "cognitive dysfunction," and the related Medical Subject Headings keywords. The keywords were searched in PubMed for all papers and studies that discussed the pathophysiology of cognitive dysfunction in fibromyalgia, its risk factors, new modalities of treatment, and other clinical implications in the past five years including full text or only abstracts (n = 29 studies). We removed any study that discussed other symptoms or complications of fibromyalgia not related to our aims and was published more than five years back (Figure 1).

## Review

Pathophysiology

How Is Brain Acting During the Higher Cognitive Function Tests in Fibromyalgia?

Besides the hippocampal atrophy and dysfunction, it was suggested that excessive consumption of neurotransmitters in the brain in response to excessive pain sensitization may lead to wasting neural resources needed for other brain functions such as cognition, and previous neuroimaging studies have shown a lower activity in brain areas that are related to the pain perception and executive functioning in fibromyalgia patients [9].

In 2017, González-Villar et al. suggested that an increase in neural noise is also responsible for dyscognition in fibromyalgia. EEG was recorded in 18 fibromyalgia (FM) patients and 22 healthy controls while performing Multi-Source Interference Task (MSIT). As expected by the authors, EEG of FM patients showed increased neuronal noise evidenced by a flatter slope of the power spectrum density and this indicates less coordination between brain areas in response to different stimuli. Modulation of theta and alpha power was less in fibromyalgia patients and midfrontal-posterior theta phase synchronization was also reduced in these patients [10].

In 2019, Samartin-Veiga et al. conducted a case-control study where 19 patients with fibromyalgia and 22 healthy controls were enrolled to evaluate the brain activity and EEG during the higher executive functions. Results showed a decrease in N3 amplitude, which indicates conflict detection and monitoring, which is important for the response program updating and successful inhibition and indicates the difficulties in processing the task stimuli. N3 wave was distributed in the frontocentral scalp with its neural basis in the dorsal portion of the anterior cingulate cortex (ACC). Reduced theta activity was also observed in FM patients than in the control group. The theta frequency is also an indicator of monitoring and response competition and the P3 wave in the temporoparietal was used to indicate a brain electrical activity in response to different trails and interference and attentional processing of the target stimuli. Although it was not confirmed that P3 has a significant reduction in FM patients than in the control group, P3 amplitude was larger in healthy controls and it was smaller in the interference condition in FM patients, which indicates a longer reaction time [9].

In 2019, Pidal-Miranda et al. conducted another study to confirm that the excessive pain sensation and processing in FM patients is associated with the allocation of attentional resources to the pain rather than other brain cognitive functions and is also associated with impairment in inhibitory processes. EEG was recorded in 31 FM patients and 28 healthy controls during performing emotional Go/No-Go task with micro-videos of pain, happy, and neutral facial expressions. The interpretation was done according to pain expression and N2, theta, delta, and P3 amplitude. It was observed that pain expression is associated with longer reaction time and errors and higher theta, delta power, and P3 amplitude to No-Go stimuli, which indicates more attention to the pain is associated with reduced performance of the inhibitory task. Although the results are similar in patients and healthy controls, N3 amplitude was modulated to be larger to pain faces only in the control group, which suggest that FM patient has difficulties in the modulation of N3 after pain faces [11].

In a similar study in the same year (2019) [12], EEG was recorded in 27 FM patients and 27 healthy controls, while performing the stop-signal paradigm (motor inhibition task). González-Villar et al. found that the FM patients have preserved mechanism of motor inhibition evidenced by similar N2, P3, and behavioral results in both groups; however, reduced modulation of posterior alpha power after go or stop stimuli was observed in FM patients, and the authors suggest FM patients have difficulties in mobilization and preserving visual attentional resources, which in turn explains the cognitive impairment in those patients [12].

Earlier in 2018, Chou et al. had also found a reduced frontal activity, which is the main area for higher mental functions during verbal fluency test (VFT), using near-infrared spectroscopy. The study was conducted on 11 FM patients and 13 healthy controls, and near-infrared spectroscopy was recorded during performing VFT, and the assessment was targeted to frontal-bilateral inferior frontal gyrus and temporal [13].

Patients with fibromyalgia have reduced pain thresholds and defective inhibitory modulation of nociceptive input. According to the previous studies that were collected in a structured review, EEG and functional magnetic resonance imaging demonstrate increased activity in cortical regions of the pain perception and processing like medial frontal, insular areas, sensory and motor cortex, and cerebellum, while decreased activity was noted in areas of inhibitory control like rostral anterior cingulate regions. Moreover, the abnormalities in the default mode network that affect the executive control network were also shown [14].

Somatosensory temporal discrimination threshold (STDT) was assessed by Gunendi et al. in 15 fibromyalgia patients compared to 15 healthy controls. STDT is defined as the shortest time interval necessary for a pair of tactile stimuli to be perceived as separate, and authors found higher somatosensory temporal discrimination threshold in FM patients and strong relation with pain intensity scores and symptoms severity scale scores [15,16].

Impairment in somatosensory temporal discrimination ability reflects the impairment in higher cognition sensory processing. All these findings suggest that FM patients have difficulties in attentional processing and modulating their responses according to the task demand as well as longer reaction time, and these studies explain how the brain is acting during complex interference tasks in FM patients. These findings reflect a reduction in frontal brain activity and augment the hypothesis of consumption of neuronal and attentional resources to the pain and reduce its availability and neutral facial expression to drive other cognitive responses to each task.

Most of these studies are case-control observational studies, related to humans, and it needs more evidence-based studies to confirm the results. All of the studies have a small number of patients ranging from 11 to 31 patients, and only one study was a structured review, which is more accurate and has more evidenced information, and has collected 16 studies.

All of the included studies are of good quality using the Newcastle-Ottawa quality assessment tool for case-control studies and Assessment of Multiple Systematic Reviews (AMSTAR) checklist for structured review, except two studies, which were unable to assess the quality because they are available as abstract [12-13].

Role of Stress Maladaptation and Level of Cortisone in Pathophysiology

It was supposed that the development of fibromyalgia is triggered by stressful conditions. Previous studies had investigated how the stress hormones level such as cortisone is related to other symptoms of fibromyalgia and decreased level of cortisone was found in many other conditions related to stress like post-traumatic stress disorder, chronic fatigue syndrome as well as fibromyalgia, and this was explained by severe or chronic stress exposure that leads to stress maladaptation.

In 2018, one study conducted by Lin et al. assessed the level of cortisone and its relation to cognitive function in fibromyalgia patients. A total of 44 FM patients were compared to 48 healthy controls in terms of subjective cognitive complaints and objective cognitive function testing of memory, language, executive functions, and diurnal level of salivary cortisol (samples were taken at awakening, 30 minutes later, 3 pm, and bedtime). Results showed FM group had more subjective cognitive complaints and poorer objective cognitive performance and the cortisone levels were lower in the FM group at 30 min after awakening with a large effect size (FM: 0.209 ± 0.145, controls: 0.386 ± 0.243 pg/mL, P = 0.001, d = 0.890) and at bedtime with a medium effect size (FM: 0.002 ± 0.018, controls: 0.024 ± 0.024 pg/mL, P = 0.021, d = 0.568) but not at awakening (FM: 0.154 ± 0.117, controls: 0.212 ± 0.175 pg/mL, P = 0.148, d = 0.392) and 3 pm (FM: 0.067 ± 0.067, controls: 0.081 ± 0.080 pg/mL, P = 0.503, d = 0.190), which raise the link between stress maladaptation and dyscognition [6]. However, another study which was done earlier in 2018 by Barceló-Martinez et al. had shown that stress is related to memory impairment also in healthy controls like FM patients. A total of 30 FM patients and 30 healthy controls were enrolled in the study and different neuropsychological variables like attention, memory, language, visual-constructive praxis, and executive functions were assessed besides cortisone level in two blood samples in the morning and evening. FM group achieved lower scores in neurophysiological measures especially in cognitive functions, but there were no significant differences between the two groups in terms of stress and cortisone levels. There was a strong relation between memory storage and flexibility of responses to each task demand in both groups. This study suggested that the greater the exposure to stress, the lower the performance in memory and cognitive response flexibility even in non-fibromyalgia subjects [17].

That means stress exposure can affect the higher cognitive and executive functions in FM and non-FM patients, although the cognitive complaints in the latter group are insignificant. This also suggests that treatment and prevention of stress might have a role in the prevention of fibromyalgia and further cognition and memory decline.

These two studies were of the case-control type, which is less evidenced and more liable to bias than randomized control trials. The first study has enrolled a larger number of patients than the second study, and both are of good quality after using the Newcastle-Ottawa quality assessment scale [6-17].

Risk factors of cognitive dysfunction in fibromyalgia

The factors that influence cognitive function in fibromyalgia were studied in 2018 [18]. Ojeda et al. have assessed the cognitive function in neuropathic pain patients, chronic pain patients, and fibromyalgia patients and compared it with healthy controls. Factors like pain, anxiety, depression, and sleep quality were studied. Cognitive performance was assessed by using the Test Your Memory (TYM) scale, pain intensity was assessed by the Visual Analog Scale (VAS), mental health was assessed by the Hospital Anxiety and Depression (HAD) scale, and sleep quality was assessed by Medical Outcome Study Sleep scale. Cognitive functions were the lowest in the fibromyalgia group. Anxiety and sleep quality have no effect on cognitive performance; however, pain intensity and depression affect cognitive functions. An increase in the HAD scale score is associated with reduced cognitive functions especially in fibromyalgia patients, also an increase in pain intensity (VAS) is also associated with reduced cognitive dysfunction in all groups compared to healthy controls. Depression was found as a confounding factor between pain and cognitive performance [18].

In another study [19], the relation between cognitive dysfunction and pain responses to low-intensity pressure stimulation, responses to stronger stimulation, pain thresholds, and intolerance were assessed in 42 women with fibromyalgia and 30 healthy women. Galvez-Sánchez et al. assessed the cognitive functions using the Spanish version of the Rey-Osterrieth Complex Figure (ROCF) tests, the verbal learning test (TAVEC), the Spanish adaptation of the Zoo Map Task (ZMT), and the Spanish version of the Trail Making Test (TMT). The pain was triggered by using a wireless pressure algometer and then pain thresholds and tolerance were evaluated. The results demonstrated an inverse association between pain intensity rating during pressure stimulation and cognitive performance in all tested cognitive domains [19].

Another study conducted by Sitges et al. has also supported the hypothesis that depression affects the information processing speed in fibromyalgia patients. A total of 17 FM patients with low depression, 18 FM patients with high depression, and 18 healthy controls were enrolled. They underwent Go trials and the inhibitory phase of the task (No-Go trials). Results showed a strong correlation between depression and affective dysregulation and slow reaction time and the authors found an inverse relationship between depression and cognitive performance [20].

Another risk of cognitive function impairment is tobacco smoking. A prospective questionnaire study was conducted by Lin et al. from May 2012 to November 2013. A total of 66 FM patients were surveyed and categorized according to their smoking status. The cognitive performance, quality of sleep, quality of life, FM symptoms severity, anxiety, and depression were compared between the subgroups. Results showed that smoking has an inverse effect on cognitive performance, worsening quality of life, increased sleep disturbance, and increased incidence of anxiety [7].

Body mass index's effect on executive functions in fibromyalgia was assessed besides other variables like pain intensity, psychiatric co-morbidity, and medications. Muñoz Ladrón de Guevara et al. assessed cognitive function using cognitive tests in 52 fibromyalgia syndrome (FMS) patients and 32 healthy controls. They found that pain intensity and high BMI have the largest proportion of reduced cognitive performance. The mean BMI in FMS was 28.9 ± 4.49 while in controls was 26.49 ± 4.36 [21]. Further studies are needed to confirm the relation between BMI and cognitive dysfunction in FM.

The above studies have demonstrated the risk factors of cognitive function decline in FM; this includes pain duration and severity, depression, tobacco smoking, and high BMI. Proper management and prevention of these factors might be helpful in cognitive dysfunction management.

To compare between these studies, four of them are of observational type case-control studies [18-21], which have less evidence and more risk of bias than randomized control trials; however, by using Newcastle-Ottawa quality assessment scale, it was found that three of them are of good quality [18-21], and the quality of the other was not assessed as it was available only as an abstract [20]. One study was a prospective questionnaire survey, which is also an observational study and has the largest number of patients (66) and also has a good quality, which is found using the same tool (Table 1) [7].

**Table 1:** Studies of risk factors of cognitive dysfunction in fibromyalgia. FM, fibromyalgia; MSK, musculoskeletal; VAS, Visual Analog Scale; HAD, Hospital Anxiety and Depression scale; BMI, body mass index.

Authors	Study design	Number of patients or studies	Risk factor	Results
Ojeda et al. [18]	Case-control study	104 patients with neuropathic pain, 99 patients with musculoskeletal pain, 51 patients with fibromyalgia, and 72 healthy subjects	Pain, depression	Cognitive performance was lower in FM patients, and depression has a negative impact on fibromyalgia patients and MSK patients. Increased VAS and HAD scores were associated with reduced cognitive performance in fibromyalgia patients
Galvez-Sánchez et al. [19]	Case-control study	42 fibromyalgia patients and 30 healthy controls	Pain	An inverse relation between pain intensity and cognitive performance was found
Sitges et al. [20]	Case-control study	17 low depression fibromyalgia patients, 18 high depression FM patients, and 18 healthy controls	Depression	An inverse relation between depression and cognitive performance was found
Ge et al. [7]	Prospective questionnaire study	66 fibromyalgia patients	Tobacco smoking	Tobacco smoking is associated with reduced cognitive performance, worsening quality of life, increased sleep disturbance and increased incidence of anxiety
Muñoz Ladrón de Guevara et al. [21]	Case-control study	52 fibromyalgia patients and 32 healthy controls	Pain, high body mass index	An inverse relation between pain intensity and BMI was found

New modalities of non-invasive brain stimulation

Management of fibromyalgia includes pharmacological and non-pharmacological treatment. Brain neurostimulation by electrical current is proposed for cognitive impairment and other fibromyalgia symptoms management. Transcranial direct-current stimulation of motor cortex M1 reduces pain and transcranial direct-current stimulation of dorsolateral prefrontal cortex improves anxiety, depression attention, memory, and cognitive impairment in FM patients [22]. In 2017, Curatolo et al. had assessed in a randomized controlled trial the effect of newer transcranial random noise stimulation (tRNS) of motor cortex M1 compared to transcranial direct-current stimulation (tDCS) of the dorsolateral prefrontal cortex [23]. A total of 20 FM females were randomized to receive active or placebo tRNS sessions. Pain and other cognitive functions were assessed before and after the sessions using the Visual Analogue Scale, Fibromyalgia Impact Questionnaire (FIQ), Hospital Anxiety and Depression Scale, the Trail Making Test, the Rey Auditory Verbal Learning Test (RAVLT), the Forward and Backward Digit Span Test, and the F-A-S verbal fluency test. The results suggested that tRNS of M1 might be very effective in relieving pain, depression, and anxiety in fibromyalgia, and a significant improvement in verbal fluency test, trail making test, FIQ, and RAVLT was also shown [23].

The effect of transcranial direct current stimulation with working memory training was assessed in a randomized controlled trial in 2018 by Santos et al. A total of 40 FM females were randomized to receive eight sessions of either active or sham (placebo) anodal transcranial direct current stimulation (tDCS). Anodal stimulation was targeted to the dorsolateral prefrontal cortex (DLPFC) combined with working memory training for 20 minutes. Neurocognitive tests were done and the results showed significant improvement in immediate memory index, an increase in orthographic and semantic verbal fluency scores, and short-term memory compared to sham [24]. According to the recent review study that was done in 2019, Brighina et al. found that the targeted stimulation to M1 using tDCS shows great efficacy in reducing pain while stimulation of DLPFC shows less efficacy in reducing pain and has limited effect on cognitive and affective symptoms. The review was also covering the possible use of affordable high-density tDCS and tRNS devices at patients' homes [14].

The new modality of non-invasive brain stimulation might be very useful for cognitive impairment and other fibromyalgia symptoms treatment, especially for cases that are not responding to pharmacological treatment. Also, it can be considered as an add-on therapy to the usual treatment.

Three of these studies were randomized control trials, which are human-related and have more evidenced information and less bias [22-24]; moreover, by using the Cochrane risk-of-bias tool, two studies were found of good quality and both have included the same number of patients [22-24]. The other study has included 20 patients but it is available as an abstract and its quality was not assessed [23]. The fourth study is a structured review, which is also a strong type and has more evidence and less bias, and it was of good quality, which is found using the AMSTAR checklist (Table 2) [14].

**Table 2:** Studies of a new modality of non-invasive brain stimulation in fibromyalgia. tDCS, transcranial direct currents stimulation; tRNS, transcranial random noise stimulation; DLPFC, dorsolateral prefrontal cortex; FIQ, Fibromyalgia Impact Questionnaire; HPth, heat pain threshold; RAVLT, Rey Auditory Verbal Learning Test; M1; motor 1 cortex.

Authors	Study design	Number of patients or studies	Aim	Results
Silva et al. [22]	Randomized controlled trial	40 patients	To assess the effect of tDCS of DLPFC	An increase in performance of orienting and executive attention network as well as an increase of pain threshold and HPth
Curatolo et al. [23]	Randomized controlled trial	20 patients	To assess the effect of tRNS of M1 on fibromyalgia symptoms	A significant improvement in verbal fluency test, Trail Making Test, FIQ, and RAVLT, and improvement of pain and depression symptoms
Santos et al. [24]	Randomized controlled trial	40 patients	To assess the effect of tDCS of DLPFC with working memory training on fibromyalgia symptoms	A significant improvement in immediate memory index and an increase in orthographic and semantic verbal fluency scores and short-term memory
Brighina et al. [14]	Structured review	16 studies	To assess the effect of various transcranial electrical stimulation on M1 and DLPFC in FM management	Targeted stimulation of M1 by tDCS has a great effect on pain reduction while stimulation of DLPFC has less effect on pain reduction and might have a limited effect to improve the cognitive dysfunction

Clinical implications of fibromyalgia and fibro fog diagnosis, treatment, and prevention

Some physicians think the subjective cognitive complaints are overstated by the patients [25]; however, many previous studies confirmed that these subjective complaints are confirmed objectively in cognitive function testing. Early detection of cognitive function decline in fibromyalgia can be helpful to patients for their daily lives and activities. Estévez-López et al. found in a case-control study that cognitive dysfunction in fibromyalgia patients can affect physical performance and increase pain catastrophizing [26], so it is wise to screen for any cognitive impairment symptoms and manage it properly to improve the overall health of FM patients and assure the maximum physical function.

Cognitive complaints can be screened during the clinical consultations by completing questionnaires, and objective assessment can be confirmed by various measures. Event-related potential (ERP) and EEG are potential tools to detect cognitive dysfunction in chronic pain syndromes including fibromyalgia, according to a recent study in 2021 [27]. Another potential tool to assess the brain activity in the frontal area is near-infrared septectomy during a verbal fluency test [13]. Neuroimaging such as magnetic resonance spectroscopy can be useful for monitoring response to pharmacological treatment in fibromyalgia [28].

It was also demonstrated that cognitive function impairment can be triggered by stress and worsened by pain experience; hence, proper management of stress and pain in fibromyalgia can improve cognitive function [18-29].

Lin et al. suggested that stress maladaptation may play a role in the development of dyscognition in fibromyalgia and they suggested for further studies to confirm if stress management may improve cognitive function [6]. This is supported by a randomized controlled trial to compare the effect of mindfulness-based stress reduction with the other usual modalities of treatment for fibromyalgia [30]. Pérez-Aranda et al. compared the treatment outcome between groups immediately after the treatment and at 12 months follow-up. Results showed that the mindfulness-based stress reduction group was superior to other groups at post-treatment time and at twelve-month follow-up in reducing the functional impairment; however, it did not include the effect on cognitive dysfunction. This study showed the impact of stress management in overall fibromyalgia symptoms improvement [30].

While depression is frequently associated with fibromyalgia, a clinical approach to detect and treat these depressive symptoms can play a great role in the improvement of cognitive performance and management of body weight, and encouraging smoking cessation also can be useful [21-27,31].

FM patients showed olfactory nerve dysfunction and this was found to be related to cognitive dysfunction. Blanco et al. conducted a case-control study with 146 FM patients and 122 healthy controls. They collected data using the Wechsler Adult Intelligence Scale III (WAIS-III; to assess cognitive functioning) and the Connecticut Chemosensory Clinical Research Center test (CCRC; to assess olfactory functioning). Results suggested that olfactory nerve dysfunction can be a predictor of cognitive decline and used as an early marker for dyscognition in fibromyalgia and other diseases like Alzheimer's and Parkinson's disease; however, more studies are required to confirm these results [32].

## Conclusions

The cognitive dysfunction in fibromyalgia might be caused by various mechanisms in the brain. It can be due to mechanisms related to the disease symptomology like excessive pain perception and can be due to mechanisms related to hormonal and metabolite changes in the brain. It was useful to find the risk factors of fibro fog to prevent the cognitive dysfunction in fibromyalgia.

This study has demonstrated the importance of screening cognitive complaints in fibromyalgia patients during consultations. Using various tools to assess the cognitive functions in FM patients with cognitive complaints was also discussed in the review. Using the recent affordable effective non-invasive brain stimulation therapy at homes can be useful and more convenient to the patients. This study is limited by less evidence-based studies. Most of the collected studies were case-control studies and they included a small number of patients. Some specific topics were discussed in a few numbers of studies, for which we recommend more studies to be conducted. The study directs the researchers to conduct more studies about the risk factors and diagnostic procedures for further development of the clinical approaches to managing fibromyalgia and fibro fog.
